# Evaluations of microvascular density by optical coherence tomography, angiography, and function by multifocal electroretinography of the macular area in eyes with branch retinal artery occlusion

**DOI:** 10.3389/fopht.2023.1255098

**Published:** 2023-11-08

**Authors:** Yuro Igawa, Haruna Amaki, Junji Kanno, Midori Tachibana, Satomi Konno, Yuji Yoshikawa, Soiti Matsumoto, Takuhei Shoji, Jun Makita, Kei Shinoda

**Affiliations:** ^1^ Department of Ophthalmology, Saitama Medical University, Faculty of Medicine, Saitama, Japan; ^2^ Department of Ophthalmology, Matsumoto Eye Clinic, Awa, Japan; ^3^ Department of Ophthalmology, Koedo Eye Institute, Kawagoei, Saitama, Japan

**Keywords:** multifocal electroretinography (mfERG), branch retinal artery occlusion (BRAO), layer-by-layer analysis, optical coherence tomography angiography (OCTA), optical coherence tomography (OCT)

## Abstract

**Introduction:**

It is reported that eyes with a branch retinal artery occlusion (BRAO) had normal full-field electroretinography (ERG) but the response of the multifocal electroretinography (mfERG) was reduced in the area of the arterial occlusion. Optical coherence tomography angiography (OCTA) is a recently appeared modality that can evaluate microvascularizations in different retinal layers and in different regions of the retina. The purpose of this study was to determine the density of the microcirculation and the function of the macular area of eyes with BRAO, and to determine whether they are significantly correlated.

**Methods:**

The OCTA and mfERG findings of 7 eyes of 6 patients (3 men, 3 women) were studied. The mean age of the patients was 71.7±10.6 years. The OCTA examinations were made with volume scans of 3 × 3 and 6 × 6 mm squares centered on the fovea. The macular vessel densities (mVD) in the superficial retinal layer (SRL) and deep retinal layer (DRL) were measured for the superior and inferior halves of 3  × 3  and 6  × 6 mm diameter concentric circles. The mfERGs were recorded with targets set to stimulate the focal areas of the retina corresponding to the areas examined by OCTA.

**Results:**

The OCTA examinations showed that the mVD of the 3 mm concentric circle in the SRL was significantly lower on the affected side than on the unaffected side (P = 0.022). No such difference was observed in the DRL. The N1 amplitude of the 20.2° concentric circle and the N1–P1 amplitude of the 10.1° concentric circle of the mfERGs were significantly smaller on the affected side than on the unaffected side (P = 0.047 and 0.031). A significant positive correlation was found between the mVD of the 6 mm concentric circle in the DRL and the P1–N2 amplitude of the 20.2° concentric circle (*ρ* = −0.929 and *p* = 0.003).

**Discussion:**

These findings indicate that OCTA images may be able to show changes in the density of the retinal macular microcirculation, and the mfERGs may be able to show alterations in the function of the macular area of the eyes with BRAO. A layer-by-layer analysis of the local retinal microcirculation and function should help in determining the pathogenesis of BRAO.

## Introduction

Optical coherence tomography angiography (OCTA) is a non-invasive technique that can obtain *en face* images of the blood vessels and microvascularizations of the retina (1–3). The images obtained by OCTA can be used to determine the pattern and density of the retinal vessels in different retinal layers and different regions of the retina (1–3). It has been shown that OCTA can reveal the extent of the macular ischemia, including perfusion defects in the radial peripapillary capillaries (4) and the superficial vascular capillary plexus (5) that cannot be seen in fluorescein angiograms (FAs). In addition, OCTA has been shown to be helpful in monitoring the vascularization of eyes with retinal artery occlusions (RAOs) (6).

Multifocal electroretinography (mfERG) is the recording of the electrophysiological responses at multiple retinal loci, including the macular area. It has been widely used to diagnose and determine the stage of various focal retinal diseases (7). Thus, Kondo et al. reported that eyes with a branch retinal artery occlusion (BRAO) had normal full-field ERGs but that the amplitudes of the mfERGs were reduced in the area of the arterial occlusion. The amplitudes of both the negative and positive waves were smaller than those of the mfERG components of normal eyes, but a negative configuration of the mfERGs was not observed in the affected area (8). Although local microcirculatory disturbances with associated local retinal dysfunction have been reported, none of the studies have examined whether these changes were significantly correlated.

Thus, the purpose of this study was to determine the significance of the correlations between the density of the microcirculation and the function of the area that is nourished by the microcirculation in eyes with BRAO. To accomplish this, we determined the density of the microcirculation of the macula area by OCTA and the physiology of the same area by mfERGs. These recordings allowed us to perform a layer-by-layer analysis of the local retinal microcirculation and function in eyes with BRAO.

## Patients and methods

The OCTA images and mfERG responses of patients diagnosed with BRAO at the Saitama Medical University Hospital between October 2021 and December 2022 were analyzed. The procedures used in this study adhered to the tenets of the Declaration of Helsinki, and the study protocol was approved by the Ethics Committee of Saitama Medical University (2022–090). The data of the patients were used only for this study, and patient confidentiality was preserved. The data sets generated and analyzed for this study are available on request.

All participants underwent comprehensive ophthalmic examinations, including measurements of the best corrected visual acuity (BCVA) with a Landolt chart, slit-lamp biomicroscopy of the anterior segment, measurements of the intraocular pressure by Goldmann applanation tonometry, and fundus photography with the CX-1 camera (Canon, Inc., Tokyo, Japan). All participants underwent swept-source OCTA (SS-OCTA; PLEX Elite 9000, version 1.6.0.21130; Carl Zeiss Meditec) and spectral-domain OCT (SD-OCT; SPECTRALIS HRA 2, Heidelberg Engineering, Heidelberg, Germany) examinations. In addition, mfERG recordings (LE-4100 system; Mayo Corporation, Inazawa, Japan) were used to determine the function of the focal areas of the macular region.

### OCT determination of macular retinal thickness

**Figure 1:**
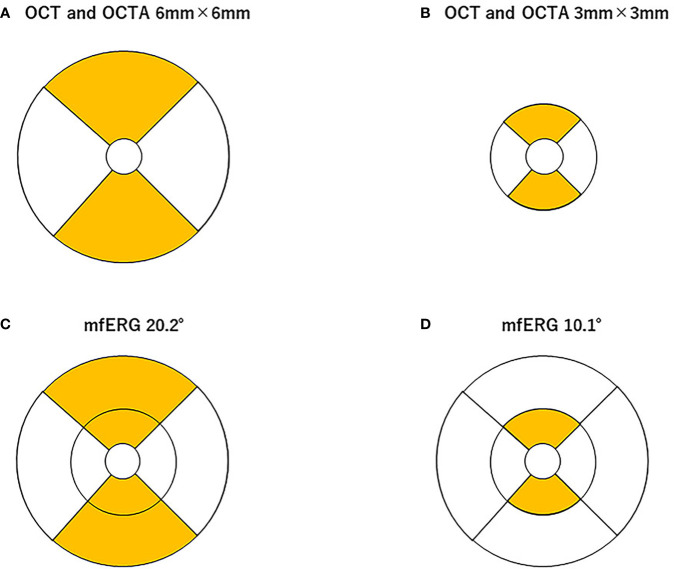
Area of analysis for optical coherence tomography (OCT), OCT angiography (OCTA), and multifocal electroretinography (mfERG). The mean retinal thickness of the 3-mm **(A)** and 6-mm **(B)** concentric circles on the affected and unaffected sides was measured using built-in software in the spectral-domain OCT. The macular vessel density (mVD) in the superficial retinal layer (SRL) and deep retinal layer (DRL) were measured in the superior and inferior halves of 3-mm and 6-mm diameter concentric circles (excluding the 1-mm diameter circle on the 3-mm and 6-mm square image), respectively **(A, B)**. These areas correspond to the areas of the OCT images. The stimuli for the mfERG were created on a digital light processing (DLP) display (Dell Projector M318WL; Dell Technologies Inc., Round Rock, TX, USA) located approximately 15.6 cm in front of the patient’s eye. The stimulus consisted of nine elements arranged in a dart pattern with an overall diameter of 3.37°, 10.1°, or 20.2°, and they were arranged to record focal responses from the retinal area corresponding to the areas of the OCTA images. The amplitude of the N1, N1–P1, and P1–N2 waves and the implicit times of the N1, P1, and N2 waves were measured in the superior and inferior halves of the 10.1°- and 20.2°-diameter concentric circles, but not the 3.7°-diameter circle **(C, D)**. The amplitudes of the N1, N1–P1, and P1–N2 and the implicit times of the N1, P1, and N2 from each area were measured.

A square pattern centered on the macula consisting of 25 parallel B-scans that were 9 mm in length was used to obtain high-resolution cross-sections of the retina using the SPECTRALIS. The light source of the SPECTRALIS has a wavelength of 880 nm and an axial resolution of 7 μm. To improve the image quality, a minimum of 40 B-scans were averaged for each cross-section image using the automatic real-time (ART) mean algorithm that is embedded in the SPECTRALIS programs. Images with a quality index of < 30 were excluded, and the scan was repeated. The entire retinal thickness, that is, the distance between the internal limiting membrane and Bruch’s membrane, was measured with built-in segmentation software. The Early Treatment Diabetic Retinopathy Study (ETDRS) grid was used, which divides the retina into three rings, that is, a central foveal ring 1 mm in diameter, an inner macular ring 3 mm in diameter, and an outer macular ring 6 mm in diameter. Each ring was divided into four quadrants, *viz*., the superior, inferior, nasal, and temporal, from which the mean retinal thickness of the 3-mm and 6-mm concentric circles on the affected and unaffected sides were obtained (**Figure 1**).

### OCTA determination of macular vessel density

All SS-OCTA volume scans were centered on the fovea and were made over an area of 6 mm × 6 mm (300 pixels × 300 pixels). The center wavelength of the light source of the SS-OCTA system used was 1,060 nm, and the A-scan rate was 100,000 scans per s. The axial and transverse tissue resolutions were 6.0 μm and 20.0 μm, respectively. The angiographic images were processed using optical microangiography, employing both phase/Doppler shift and amplitude variations (9). All OCTA *en face* images of the superficial retinal layer (SRL), which is located between the inner surface of the internal limiting membrane and the outer surface of the inner plexiform layer, were analyzed. The deep retinal layer (DRL), which is located between the inner surface of the inner plexiform layer and the outer surface of the outer plexiform layer, was also analyzed. The thickness of both layers was automatically determined using the built-in segmentation software. The OCTA images of the DRL were also analyzed using the built-in projection artifact removal software. In the eyes with BRAO, the retinal layer structures were disrupted, which could have led to segmentation errors during the OCTA analyses. To minimize this error, the segmentation was carefully checked in each image, and the images with segmentation failures, artifacts, or off-centered positioning were excluded from the analyses. **Supplementary Figures 1** and **2** show the segmentation of an OCTA image used for the analysis of each eye. The mVD of the SRL and DRL were determined for annuli with an outer diameter of 3-mm and 6-mm concentric annuli on the affected and unaffected sides, respectively (**Figure 1**). The angiographic images were examined by the Otsu analysis (10) for the OCTA images binarized by the ImageJ software of the National Institutes of Health (Bethesda, MD) to obtain the mVD. The mVD was then calculated as the percentage of the area occupied by the vessels relative to the concentric circular area. This method has been reported to have excellent reproducibility (11).

### Multifocal electroretinography

The mfERGs were recorded under room illumination when the participants’ pupils were dilated. The stimuli were presented with a digital light processing (DLP) display, and they consisted of nine elements arranged in a dart pattern with an overall diameter of 3.37°, 10.1°, or 20.2° (12–14). The individual stimulus was placed to record a focal response from the retina corresponding to the area examined by OCTA (see the ETDRS chart areas; **Figure 1**). The selected stimulated areas were also displayed on the monitor. The focal ERGs and the area stimulated are shown in **Figure 1**. The stimuli were presented in an m-sequence at a rate of 75 frames per s and a cycle of 2^13^–1 steps. The stimulus intensity was 20 cd/m^2^, with 1,500 cd/m^2^ for the white areas and 28 cd/m^2^ for the black areas. Great care was taken to ensure a steady fixation on a cross on the central stimulus element. In addition, the examiner carefully examined the fixation on a monitor screen for each recording. All recordings were monocular, and the refractive error of the eye was fully corrected. The responses were picked up by a silver plate electrode placed on the lower eyelid, amplified at × 248, and the half-amplitude bandpass filter was set to 10 Hz–60 Hz. The reference electrode was placed on the lower eyelid of the opposite eye, and the ground electrode was placed on one earlobe. The signals were digitalized at a 1200-Hz sampling frequency. The recording time was 1 min and 49 s. The amplitudes of N1, N1–P1, and P1–N2 and the implicit times of N1, P1, and N2 from each area (see **Figure 1**) were measured.

### Data analyses

Because of the small sample size of seven and the fact that not all parameters were normally distributed, the data were treated uniformly as non-parametric data and were reported as medians and quartiles. The OCT, OCTA, and mfERG components on the affected and unaffected sides were compared using the Wilcoxon signed-rank test. The correlation of the mVD of the affected side with the mfERG components recorded from the corresponding area was statistically evaluated using Spearman’s rank-order correlation coefficient. All statistical analyses were performed using JMP 10.1 (SAS Institute, Cary, NC, USA). A *p*-value of *<* 0.05 was considered statistically significant.

## Results

Seven eyes of six patients (three men and three women) with a mean age of 71.7 years ± 10.6 years [median and quartiles, 75.5 and (68.0, 77.8) years] were studied. The patients’ clinical characteristics are shown in **Table 1**. Three eyes had retinal ischemic changes in the superior, and four eyes had these in the inferior segments of the fundus of the eye (**Supplementary Figure 2**).

**Table 1:** Patients’ clinical characteristics.

Patient number			1	2	3	4	5	6	
Age (years)			66	78	74	77	82	53	
Sex			Female	Female	Male	Male	Female	Male	
Eye laterality			Left	Left	Left	Right	Right	Right	Left
Location of BRAO occurrence			Inferior	Superior	Inferior	Inferior	Inferior	Inferior	Superior
Visual acuity (log-MAR)			0.10	0.30	−0.08	0.22	0.40	−0.08	−0.08
Intraocular pressure (mmHg)			16	13	16	13	16	16	16
Refraction (diopter)			0.75	0.00	0.50	0.25	−0.50	1.50	1.75
Lens status			Cataract	IOL	IOL	Cataract	IOL	Cataract	Cataract
Axial length (mm)			–	21.9	24.5	26.0	23.7	–	–
Retinal thickness (μm)	Whole		–	327	328	270	251	230	234
	3-mm diameter, except 1 mm on center*	Superior	–	456	322	326	297	289	318
		Inferior	–	317	379	377	348	288	290
	6-mm diameter, except 1 mm on center*	Superior	–	408	212	288	286	277	317
		Inferior	–	297	317	308	301	266	254
Systemic diseases			–	HT, DM	Carcinoma prostate	HT, HL, DM, colorectal cancer, and heart failure	HT	HT, heart failure, and cerebral infarction	
Medications administered			–	–	–	DPP-4 inhibitors	PGF2α	CCB	
						Biguanides, CCB	Alpha-2 agonists	Anti-aldosterone drug	
						Anti-aldosterone drug	CAI, biazpirin		
Duration from symptom onset to examination (days)			1	32	6	35	6	18	Uncertain

BRAO, Branch Retinal Artery Occlusion; IOL, intraocular lens; HT, hypertension; DM, diabetes mellitus; HL, hyperlipemia; NTG, normal tension glaucoma; DPP-4, dipeptidyl peptidase 4; CCB, carbonic anhydrase; PGF2α; prostaglandin 2α; CAI, carbonic anhydrase inhibitor. *The mean retinal thickness of the 6-mm concentric circle on the affected side was significantly larger than that on the unaffected side (p = 0.0303), while no significant difference was found between the affected and unaffected side in the mean retinal thickness of the 3-mm concentric circle (p = 0.1282).

### OCT findings

The mean retinal thickness of the 6-mm concentric circle on the affected side was significantly larger than that of the unaffected side. No significant difference was found between the affected and unaffected sides in terms of the mean retinal thickness of the 3-mm concentric circle (**Table 1**).

### OCTA findings

Microvascular dropout in the retinal ischemic area was observed in the OCTA *en face* images (**Supplementary Figure 3**). The mVD of the 3-mm concentric circle in the SRL was significantly lower on the affected side than on the unaffected side (*p* = 0.022; **Table 2**). All other comparisons of the mVD on the affected and unaffected sides were not significantly different (**Table 2**).

**Table 2:** The microvessel density measured using OCTA and the amplitude and latency of each component recorded using mfERG.

			3 × 3				6 × 6			
			Affected	Unaffected	*p-value*	A-to-U ratio	Affected	Unaffected	*p-value*	A-to-U ratio
OCTA	mVD in SRL (%)		31.5 (17.2, 32.3)	34.6 (28.8, 38.5)	0.443	0.83 (0.45, 0.95)	41.8 (29.8, 43.6)	41.2 (40.6, 42.2)	1	1.01 (0.71, 1.07)
	mVD in DRL (%)		14.3 (6.3, 18.5)	30.8 (22.6, 32.1)	0.022	0.44 (0.21, 0.90)	39.2 (27.7, 41.6)	39.6 (35.6, 48.1)	0.371	1.07 (0.58, 1.13)
			10.1°				20.2°			
			Affected	Unaffected	*p-value*	A-to-U ratio	Affected	Unaffected	*p-value*	A-to-U ratio
mfERG	Amplitude (nV/deg2)	N1	−6.7 (−7.1, −5.1)	−6.5 (−10.0, −5.7)	0.813	1.12 (0.72, 1.25)	−3.2 (−4.4, −1.9)	−4.3 (−5.0, −3.5)	0.047	0.66 (0.53, 0.89)
		N1P1	12.0 (10.6, 20.0)	13.7 (11.7, 25.8)	0.031	0.83 (0.77, 0.90)	5.4 (5.2, 6.8)	10.0 (8.6, 11.6)	0.109	0.54 (0.46, 0.79)
		P1N2	−14.2 (−18.0, −3.81)	−16.1 (−21.6, −8.5)	0.297	0.63 (0.46, 0.82)	−6.3 (−8.2, −2.7)	−8.4 (−10.5, −5.6)	0.375	0.63 (0.42, 1.11)
	Latency (m/sec)	N1	15.2 (14.7, 18.3)	15.7 (15.5, 17.5)	0.938	0.98 (0.80, 1.17)	16.5 (15.6, 17.0)	15.7 (15.4, 16.9)	0.688	1.04 (0.84, 1.08
		P1	30.5 (30.0, 33.4)	31.0 (28.0, 33.0)	0.93	1.03 (0.88, 1.09)	31.0 (29.0, 32.1)	30.4 (28.5, 31.2)	0.813	1.06 (0.95, 1.08)
		N2	44.3 (38.7, 48.5)	42.9 (41.7, 45.3)	1	1.00 (0.88, 1.07)	42.0 (40.2, 45.6)	44.5 (44.0, 47.0)	0.219	0.91 (0.83, 1.04)

The values are shown as medians (quartiles).

OCTA, optical coherence tomography angiography; mfERG, multifocal electroretinogram; mVD, microvessel density; SRL, superficial retinal layer; DRL, deep retinal layer. A-to-U ratio is the ratio of the affected side to the unaffected side.

*: Values are shown as median (quartiles). P values less than 0.05 are shaded.

### Multifocal electroretinography

The amplitude of N1 of the 20.2°-diameter concentric circle was significantly smaller on the affected side than on the unaffected corresponding area (*p* = 0.047; **Table 2**). The amplitude of N1–P1 of the 10.1° concentric circle was significantly smaller on the affected side than on the unaffected side (*p* = 0.031; **Table 2**). All other comparisons of mfERG components on the affected and unaffected sides were not significantly different (**Table 2**).

### Correlations between OCTA and mfERGs

**Figure 2:**
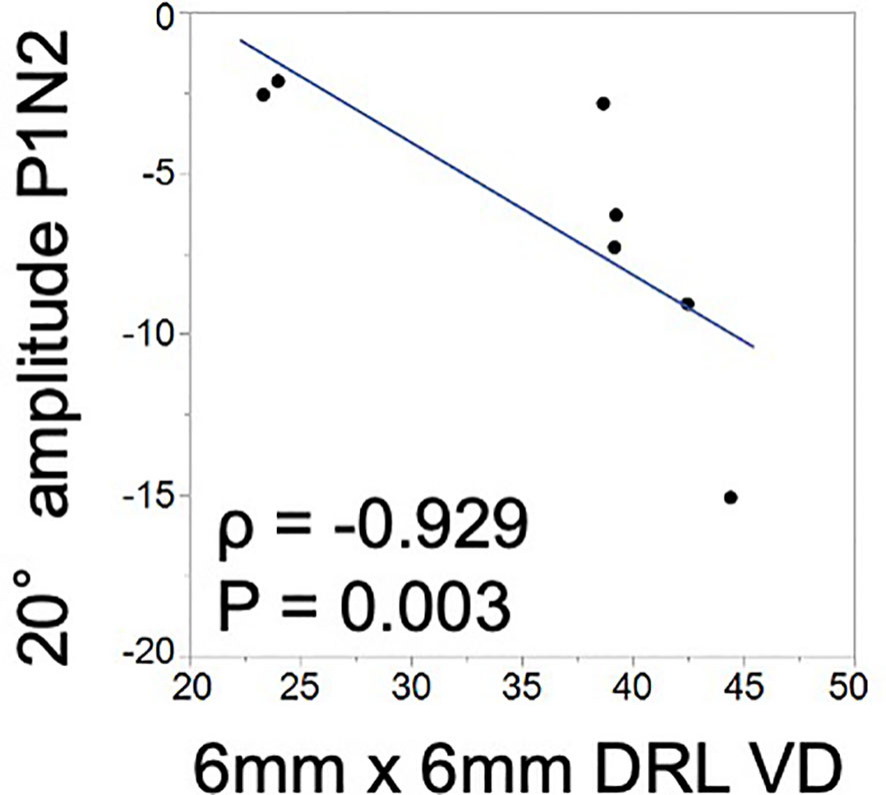
Relationship between the macular vessel density (mVD) and multifocal electroretinography (mfERG) parameters. There is a significant positive correlation between the macular vessel density (mVD) of the 6-mm concentric circle in the DRL and the P1–N2 amplitude of the 20.2° concentric circle (ρ = −0.929 and *p* = 0.003).

The mVD of the 6-mm concentric circle in the DRL was significantly and negatively correlated with the amplitude of P1–N2 of the 20.2° (*ρ* = −0.929, *p* = 0.003; **Figure 2**; **Table 3**). This suggested that the amplitude of the P1–N2 component was larger at a higher mVD.

**Table 3:** Correlation between the macular vessel density (mVD) and the multifocal electroretinogram (mfERG) parameter.

OCTA 3 mm × 3 mm vs mfERG 10.1°						
	Amplitude			Latency		
	N1	N1P1	P1N2	N1	P1	N2
SRL VD	0.429, 0.337	0.250, 0.589	−0.571, 0.180	−0.750, 0.052	−0.393, 0.383	0.071, 0.879
DRL VD	0.393, 0.383	−0.179, 0.702	−0.429, 0.337	−0.464, 0.294	−0.286, 0.535	0.179, 0.702
OCTA 6 mm × 6 mm vs mfERG 20.2°						
	Amplitude			Latency		
	N1	N1P1	P1N2	N1	P1	N2
SRL VD	0.536, 0.215	−0.107, 0.819	−0.643, 0.119	−0.036, 0.939	0.321, 0.482	0.739, 0.058
DRL VD	0.179, 0.702	0.143, 0.760	**−0.929, 0.003**	−0.357, 0.432	0.250, 0.589	0.721, 0.068

mVD, microvessel density; mfERG, multifocal electroretinography; OCTA, optical coherence tomography angiography; SRL, superficial retinal layer; DRL, deep retinal layer.

The correlations were evaluated using Spearman's rank-order correlation coefficient. The values are shown as (ρ, P value).

P values less than 0.05 are shaded.

**Figure 3:**
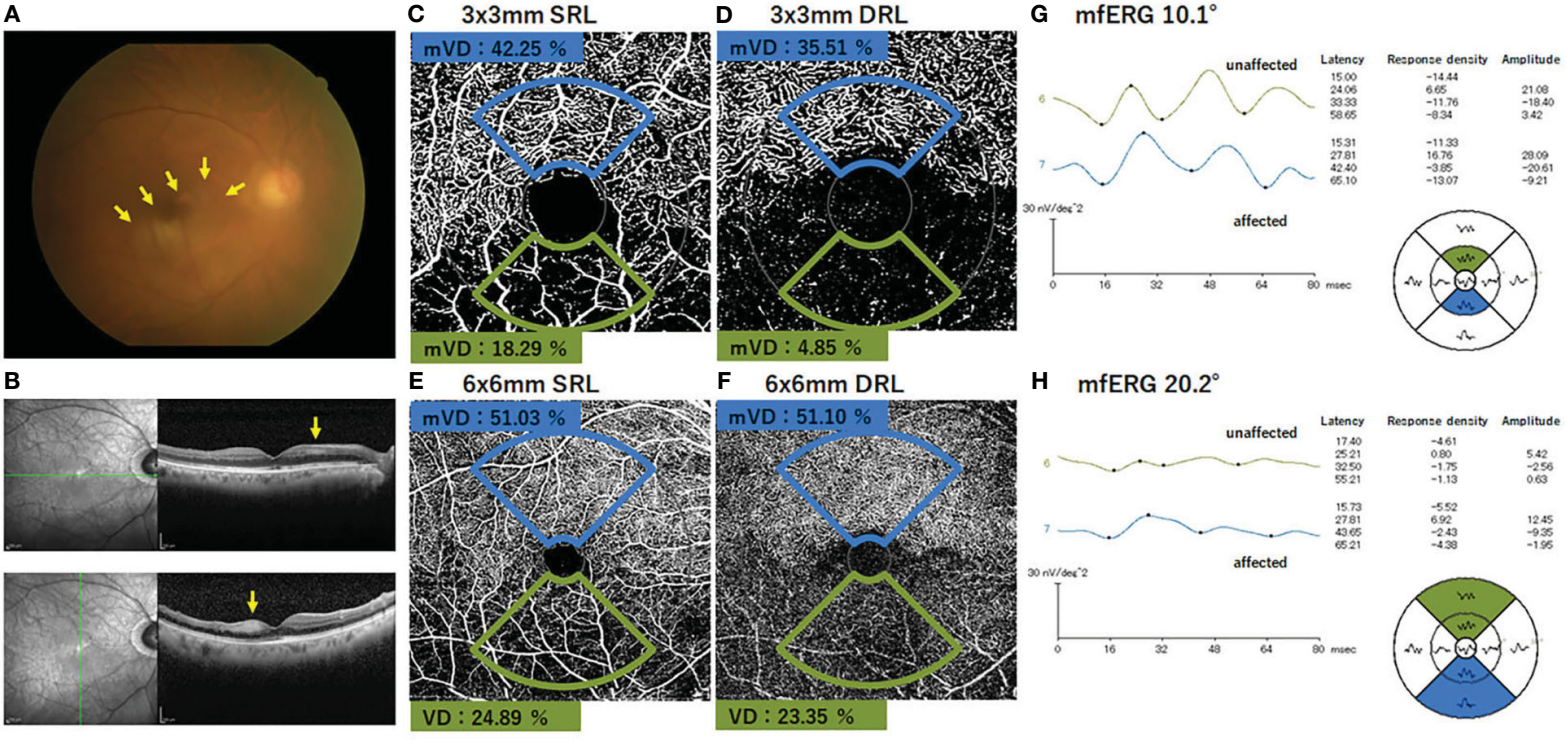
Findings in a representative case of an inferior branch retinal artery occlusion. **(A)** Fundus photograph of the right eye with inferior branch retinal artery occlusion. The retinal whitening surrounding the occluded artery can be seen (arrows). The SD-OCT images show that the inner retinal layer is thicker and more hyperreflective on the affected area than on the normal area (arrows). **(B)** OCTA image shows the presence of flow in the superior artery and capillary bed, whereas no flow signal is evident in the inferior artery and capillary. **(C–F)** The macular vessel density (mVD) was significantly lower on the affected side than on the unaffected side for both the SRL and DRL in the 3 mm × 3 mm and 6 mm × 6 mm areas. **(G–H)** The amplitudes of N1, N1–P1, and N2 were smaller on the affected side than the unaffected side for both 10.1° and 20.2° stimuli. The response in each area is shown in the field view. Therefore, the position of each waveform is diametrically reversed from the corresponding position on the fundus photograph and the OCTA image. mVD, macular vessel density; SRL, superficial retinal layer; DRL, deep retinal layer.

The fundus photographs, OCTA images, and the mfERGs of a representative case are shown in **Figure 3**. In addition, **Supplementary Figure 3** shows the images of all cases, which may be helpful in understanding the involvement of the location of the occlusion site and image distortions due to fixation loss, although the sample size was small.

## Discussion

Eyes with BRAO have different degrees of ischemic damage to the retina, which is dependent on the duration of the vascular obstruction and the degree of blood flow impairment (4–6, 15, 16). It has been reported that the degree of capillary non-perfusion detected using OCTA is correlated with the degree of retinal ischemic damage in the microvascular structure in the OCT images (17). Our results showed that the mVD in the OCTA findings of our patients with an acute BRAO was significantly correlated with the local electrophysiological findings at the site of the retinal lesion.

Qualitatively, the non-perfused areas of the SCP and DCP corresponded with the inner retinal ischemic damage as detected in the OCT *en face* images and the appearance of the fundus photographs (**Figure 3**). Although it appeared that the OCTA images clearly showed the region of vascular occlusion (**Supplementary Figure 3**), the mVD on the affected side was not significantly different from that on the unaffected side. We suggest that this discrepancy was because the technique to determine the mVD was not sensitive enough to detect the small differences, and the noise signals in the OCTA images may have hindered the accurate measurement of the affected capillary area. Thus, another binarization method is needed to optimize vessel detection more accurately.

The affected area can extend vertically to the hemisphere opposite the arterial occlusion. In such eyes, the arterial occlusion may extend both superiorly and inferiorly in a symmetrical way. A better selection of the mVD measurement areas will be helpful in obtaining a more accurate quantification of the capillary defects in eyes with BRAO.

Another concern of this study was that all the sources of noise could not be excluded by the binarization method used, and some signals were interpreted as real vascular signals. Thus, the algorithm might not have accurately identified the blood vessels in the OCTA images, and improvements are needed. However, it is known that OCTA measurements can be affected by various factors, such as the axial length, refractive error, and age (18, 19), and our study compared the symmetrical retinal areas of the same eye. Thus, the effects of these factors were probably minor.

Because a BRAO mainly affects the inner retina, the mixed rod and cone ERGs in these eyes should have a normal a-wave that arises from the photoreceptors and a reduced b-wave that originates from the bipolar and/or Mueller cells located in the inner retina (20, 21). However, the a- and b-waves of the photopic ERGs originate in part from the inner retina (22, 23), and eyes with severe CRAO exhibit a significant decrease in the amplitudes and a significant increase in the implicit times of the a-, b-, and d-waves of the conventional ERGs (24). Conversely, the results of earlier studies and those of our studies indicated that the amplitudes of the mfERGs from the affected area were smaller and the implicit times were prolonged for the N1, P1, and N2 components in eyes with BRAO. However, the mfERGs in this study did not have a negative configuration (8, 25). It has been reported that the N1 and P1 of the mfERGs are generated by the same cells that generate the a-wave and the positive peaks of the full-field photopic ERGs (26) and that while N1 originates from the cone photoreceptors, hyperpolarizing bipolar cells, and Mueller cells, P1 originates from the depolarizing bipolar cells, hyperpolarizing bipolar cells, and Mueller cells (27, 28). These sources of the mfERG components were supported by our findings.

The assessment of the function of the non-perfused area by mfERGs showed that the amplitudes of the affected side were smaller than those of the unaffected side for both the 20.2° and 10.1° concentric circular areas. These findings are in good agreement with earlier reports indicating that the reduced responses correspond to the area of arterial occlusion (8). Another study reported that the later part of the waveform of mfERGs reflects the condition of the inner retina, and the damage to the inner retina affected the N1, P1, and N2 of the first-order kernel responses (20). In addition, N2 was the most severely affected wave, which is supported by our findings.

The amplitude of P1–N2, which originates from the inner retinal layers, and the mVD of the DRL were significantly correlated (**Figure 2**). This suggested that the inner retina was damaged, which was most likely due to the microcirculation disturbances. To the best of our knowledge, this is the first study to find a significant correlation between quantitatively determined circulatory and functional alterations in eyes with BRAO. Thus, the OCTA and mfERG findings provided complementary information, and a combination of these two measures could be a good method for assessing focal retinal pathology in a layer-by-layer way in eyes with BRAO.

This study has several limitations. First, the number of participants was small because of the relative rarity of BRAOs. This resulted in relatively weak statistical power. Second, this was a cross-sectional study, and because the retinal circulation, ultrastructure, and function can change with time, the OCTA and mfERG findings can be clinically useful for monitoring the course of the disease process (6). Third, the areas examined by OCTA and mfERG did not completely correspond to the area of arterial non-perfusion. There were great variations in the extent of the BRAO lesions, and it was difficult to identify an affected area clearly. Depending on the case, the size of the lesion may be biased toward the temporal or nasal side or may be clearly divided into upper and lower regions. Therefore, it is not appropriate to perform analysis using a uniform segmentation template. This may be one reason why no significant difference was observed between the affected side and the unaffected side in the mVD analysis results using OCTA or the local function analysis results using mfERGs. These points indicate the potential errors in the methodology that should be considered for future studies. A more accurate evaluation could be obtained if the OCTA could identify the area of arterial occlusion more accurately. If this were the case, the assessment of the local retinal response with mfERGs could be focused on the corresponding area. A more detailed longitudinal observation through a combination of OCTA and mfEGs of the lesion is necessary for clinical practice, which may also be important for understanding the pathophysiology of BRAO.

## Conclusion

Our findings demonstrated that OCTA may be able to detect retinal microcirculatory disturbances and that mfERGs may be able to detect functional alterations in the macular areas in eyes with BRAO. In addition, OCTA can determine the status of the vascularization in the middle and inner retinal layers in the areas affected by BRAO. A correlation of the decreased mVD in the DRL with the attenuation of the inner retinal responses was observed. A layer-by-layer analysis of the local retinal microcirculation and function may improve our understanding of the disease course and pathophysiology of eyes with BRAO.

## Data availability statement

The original contributions presented in the study are included in the article/**Supplementary Material**. Further inquiries can be directed to the corresponding authors.

## Ethics statement

The studies involving humans were approved by the Ethics Committee of Saitama Medical University (2022-090). The studies were conducted in accordance with local legislation and institutional requirements. Patient confidentiality was preserved, and because the study was conducted in a retrospective manner, the ethics committee decided that written informed consent was waived. Written informed consent was not obtained from the individual(s) for the publication of any potentially identifiable images or data included in this article.

## Author contributions

YI: Validation, Data curation, Resources, Writing – review & editing, Investigation. HA: Validation, Writing – review & editing, Formal Analysis, Investigation. JK: Data curation, Formal Analysis, Investigation, Validation, Visualization, Writing – original draft. MT: Data curation, Resources, Validation, Writing – review & editing. SK: Investigation, Validation, Writing – review & editing. YY: Investigation, Validation, Writing – review & editing. SM: Investigation, Validation, Writing – review & editing, Conceptualization, Supervision. TS: Investigation, Validation, Writing – review & editing, Conceptualization, Formal Analysis, Methodology, Supervision. JM: Investigation, Validation, Writing – review & editing, Resources, Supervision. KS: Conceptualization, Investigation, Validation, Formal Analysis, Funding acquisition, Methodology, Project administration, Visualization, Writing – original draft.

## Supplementary material

Supplementary Figure 1 OCTA images showing segmentations for the superficial retinal layer and the deep retinal layer on the 3 mm × 3 mm macula area by means of the built-in software.

Supplementary Figure 2 OCTA images showing segmentations for the superficial retinal layer and the deep retinal layer on the 6 mm × 6 mm macula area by means of the built-in software.

Supplementary Figure 3 Fundus photograph and optical coherence tomography angiographic (OCTA) images of all eyes. Three eyes exhibited retinal ischemic changes on the superior side, and four eyes exhibited them on the inferior side, in addition to microvascular dropout corresponding to the retinal ischemic side.
