# Supplementary figures and images for: Evaluations of microvascular density by optical coherence tomography, angiography, and function by multifocal electroretinography of the macular area in eyes with branch retinal artery occlusion
Source: Front Ophthalmol (Lausanne). 2023 Nov 8;3:1255098. doi: 10.3389/fopht.2023.1255098 (PMC11182117; doi:10.3389/fopht.2023.1255098)

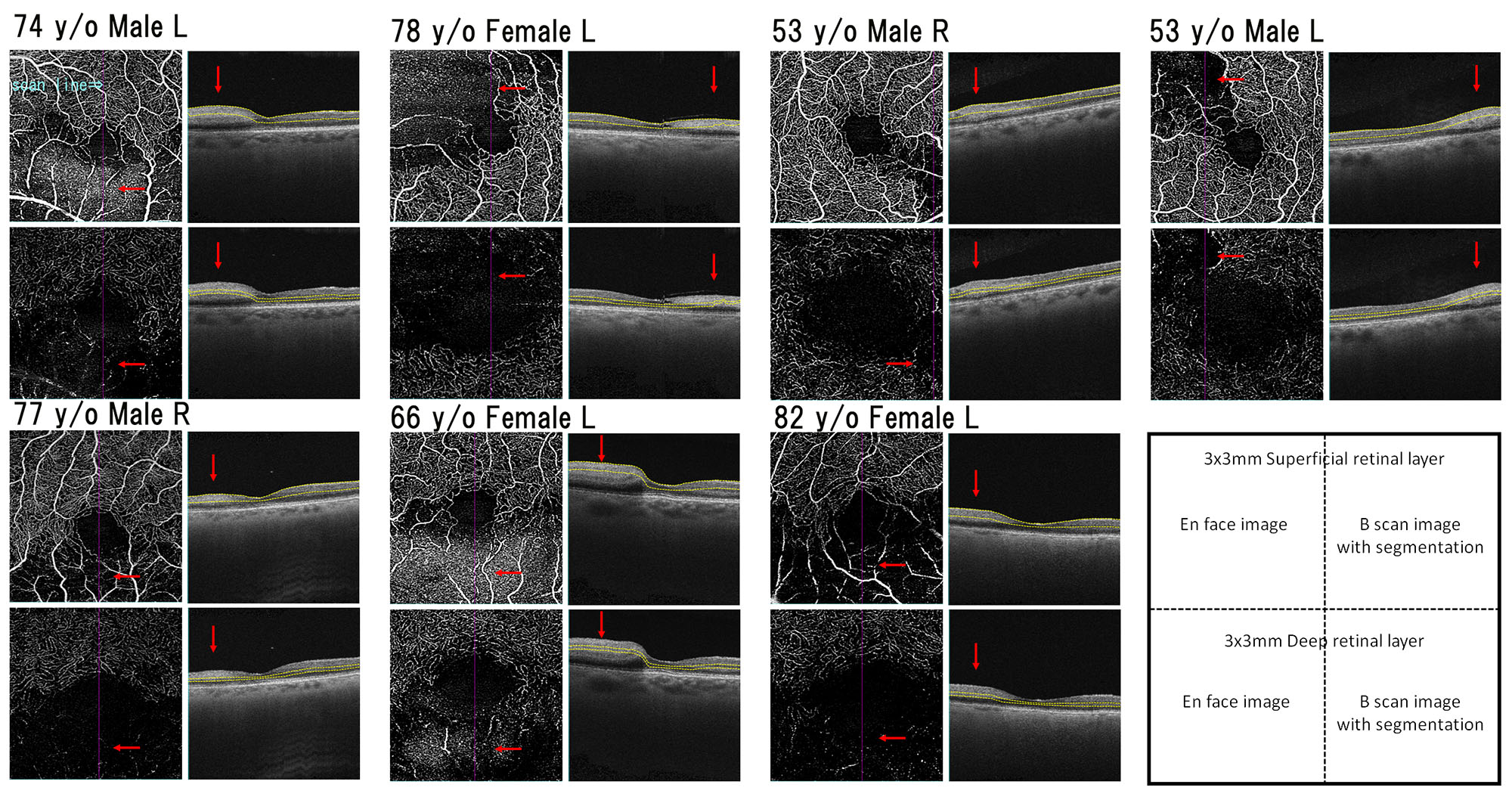

Supplement: Supplementary Figure 1 — OCTA images showing segmentations for the superficial retinal layer and the deep retinal layer on the 3 mm × 3 mm macula area by means of the built-in software. [file Image_1.jpeg]

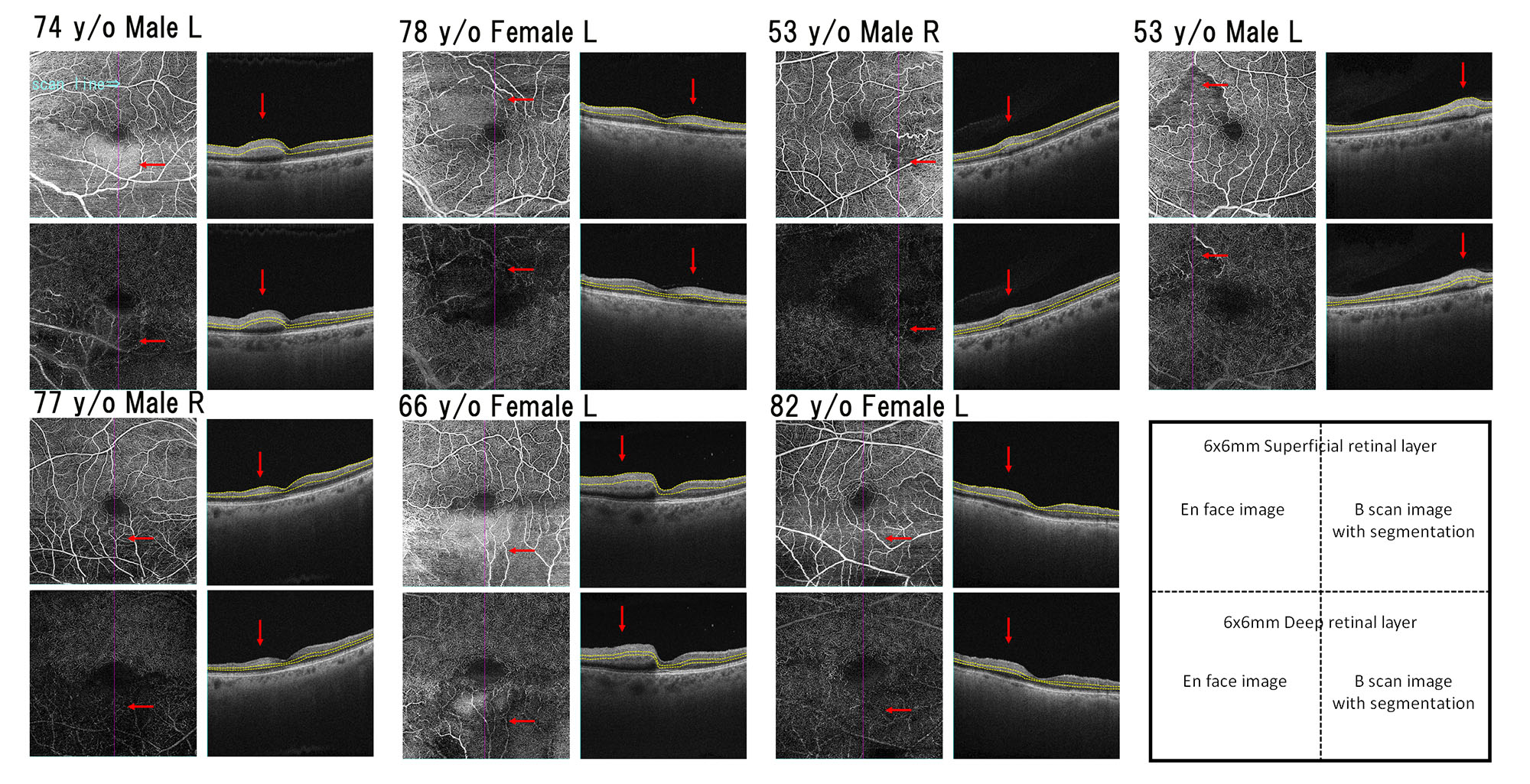

Supplement: Supplementary Figure 2 — OCTA images showing segmentations for the superficial retinal layer and the deep retinal layer on the 6 mm × 6 mm macula area by means of the built-in software. [file Image_2.jpeg]

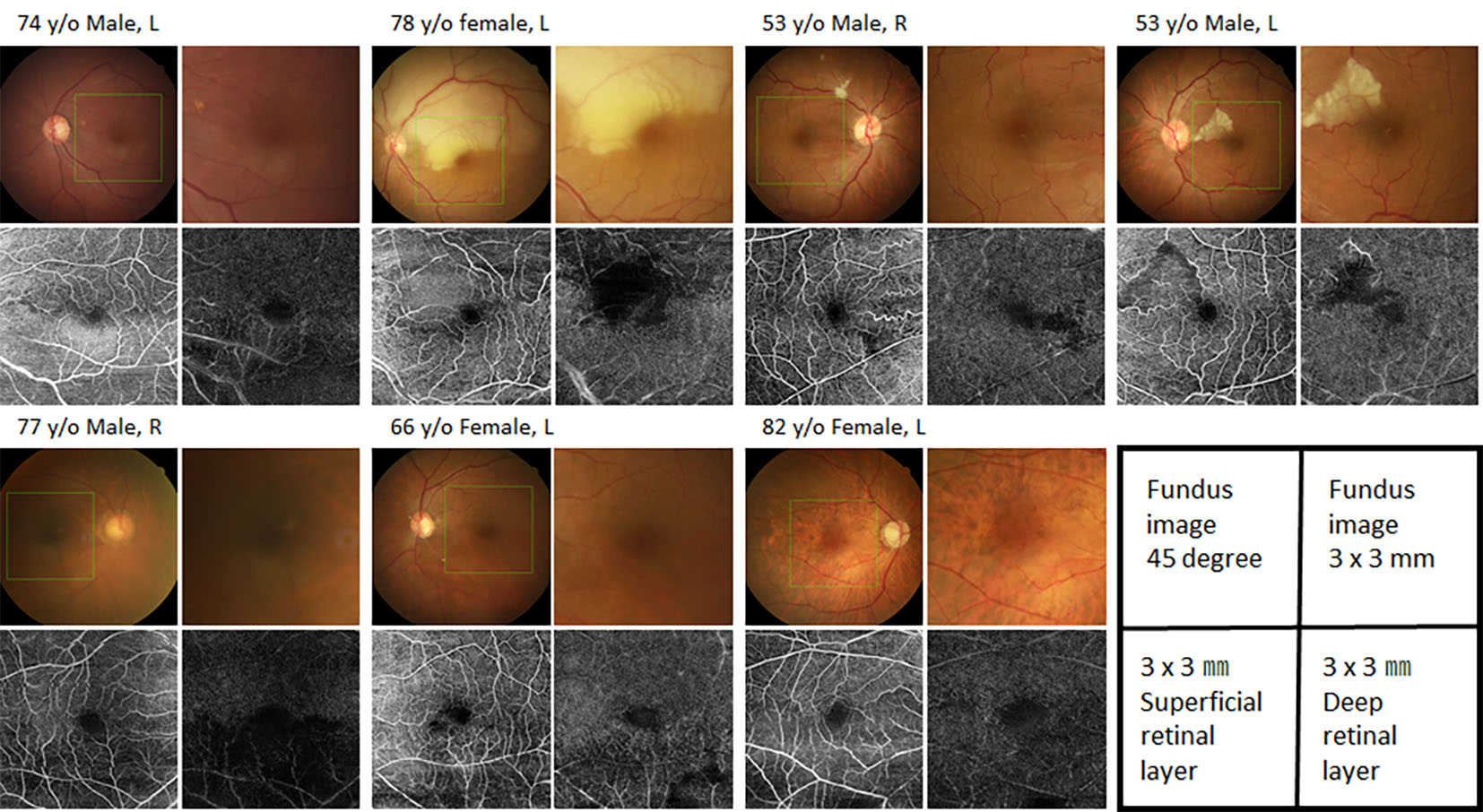

Supplement: Supplementary Figure 3 — Fundus photograph and optical coherence tomography angiographic (OCTA) images of all eyes. Three eyes exhibited retinal ischemic changes on the superior side, and four eyes exhibited them on the inferior side, in addition to microvascular dropout corresponding to the retinal ischemic side. [file Image_3.jpeg]
